# Strong selection on mandible and nest features in a carpenter bee that nests in two sympatric host plants

**DOI:** 10.1002/ece3.995

**Published:** 2014-04-17

**Authors:** Luis Flores-Prado, Carlos F Pinto, Alejandra Rojas, Francisco E Fontúrbel

**Affiliations:** 1 Instituto de Entomología, Universidad Metropolitana de Ciencias de la EducaciónAv. José Pedro Alessandri 774, Santiago, Chile; 2 Laboratorio de Química Ecológica, Facultad de Ciencias, Universidad de ChileLas Palmeras 3425, Santiago, Chile; 3 Laboratorio de Ecología Química, Facultad de Ciencias y Tecnología, Universidad Mayor de San SimónParque La Torre # 1720, Cochabamba, Bolivia; 4 Departamento de Ciencias Ecológicas, Facultad de Ciencias, Universidad de ChileLas Palmeras 3425, Santiago, Chile

**Keywords:** Fitness, nest architecture, nesting substrate, plant–insect interactions, selection gradients

## Abstract

Host plants are used by herbivorous insects as feeding or nesting resources. In wood-boring insects, host plants features may impose selective forces leading to phenotypic differentiation on traits related to nest construction. Carpenter bees build their nests in dead stems or dry twigs of shrubs and trees; thus, mandibles are essential for the nesting process, and the nest is required for egg laying and offspring survival. We explored the shape and intensity of natural selection on phenotypic variation on three size measures of the bees (intertegular width, wing length, and mandible area) and two nest architecture measures (tunnel length and diameter) on bees using the native species *Chusquea quila* (Poaceae), and the alloctonous species *Rubus ulmifolius* (Rosaceae), in central Chile. Our results showed significant and positive linear selection gradients for tunnel length on both hosts, indicating that bees building long nests have more offspring. Bees with broader mandibles show greater fitness on *C. quila* but not on *R. ulmifolius*. Considering that *C. quila* represents a selective force on mandible area, we hypothesized a high adaptive value of this trait, resulting in higher fitness values when nesting on this host, despite its wood is denser and hence more difficult to be bored.

## Introduction

Host plants can be used by herbivorous insects as food sources or primary habitats. Those plant–insect interactions could be driving local adaptations through natural selection, leading to phenotypic and genetic differentiation associated with the use of different sympatric hosts, (Berlocher and Feder 2002; Stireman et al. 2005). Also, the implications of host-mediated differentiation for insect diversification, under an ecological speciation scenario, have broadly been documented (Stireman et al. 2005). Many reports have addressed the study of ecological processes governing the rate and extent of diversification among insects exhibiting high diversity, such as some parasitic (Wiegmann et al. 1993) and phytophagous insects (Berlocher and Feder 2002), characterized by microhabitat specialization. Contrastingly, research on host-mediated differentiation and the role of natural selection on phenotypic traits directly involved in using different hosts is scarce on wood-boring insects.

Wood boring is performed in many insect groups, either to obtain food or to construct breeding sites (i.e., microhabitats where immature individuals develop). Carpenter bees of the Megachilidae (O'Toole and Raw 1991) and Apidae (Michener 2007) families are wood-boring insects which build their nests in dead stems of shrubs and trees. Nests are built only by females. They consist of simple or branched burrows, with galleries transversely divided by partitions forming a series of cells in which a food mass elaborated with pollen and nectar is deposited and used as oviposition substrate. Therefore, a nest is formed by the sequential repetition of cell construction, food mass deposition, oviposition, and cell closure (reviewed in Michener 1969, 1985, 1990).

Considering that mandibles are extensively used by carpenter bees to excavate wood and construct their nests, morphological features of this trait may be associated with the particular host species used for nesting. Thus, mandibles show some differences among bee species that manipulate different nest materials (Williams and Goodell 2000). Additionally, substrates used for nest construction are limited by the morphology of the female mandible (Hurd 1978). Thus, given the critical role of mandibles in nest construction, selection on mandible shape associated with materials used in nesting has been hypothesized, nevertheless, until now no clear adaptive significance with regard to host exploitation has been proved (Williams and Goodell 2000). On the other hand, nests are required for oviposition and immature developing, affecting fitness directly (Wilson 1971; O'Neill 2001). For example, nest length and the number of developing individuals inside them were positively correlated in a carpenter bee species, implying that larger nests bear more progeny (Flores-Prado and Niemeyer 2012).

Several studies have explored host-plant specificity in relation to nesting in carpenter bees (Díaz and Sánchez 1998; Ramalho et al. 2004; Bernardino and Gaglianone 2008); however, to the best of our knowledge, no study has addressed the role of natural selection acting upon phenotypic variation in traits directly related to fitness (e.g., mandible size and nest architecture) on carpenter bee species that use different host-plant species for nesting. In this study, we have explored both shape and intensity of natural selection on phenotypic variation upon such traits in *Manuelia postica* (Xylocopinae: Apidae), a carpenter bee species belonging to a relict genus (Daly et al. 1987), whose geographic distribution is predominantly restricted to Chile (Michener 2007). *Manuelia postica* has been found nesting in three plant species, the alloctonous shrub *Rubus ulmifolius* (Rosaceae), the native bamboo *Chusquea quila* (Poaceae), and the native tree *Aristotelia chilensis* (Elaeocarpaceae; Flores-Prado et al. 2008). There is no published data reporting other plant species used for nesting by this species. Within this context, we have examined the shape and intensity of selection gradients related to bee and nest features, which are linked with *M. postica*'s reproductive success in two co-occurring nesting hosts (*C. quila* and *R. ulmifolius*). We hypothesized that mechanical barriers imposed by host plants would be related to phenotypic traits associated with nest building, which would be mediated by natural selection on two sympatric host plants.

## Materials and Methods

### Study area and data collection

Nests were collected near to Altos de Lircay National Park, central Chile (35°29′S; 70°58′W), during austral summer and fall (February–April) periods during 2012 and 2013, corresponding to the end of the yearly breeding stages (Flores-Prado et al. 2008). This approach ensured that individuals in the nest have completed their development and reached the adult stage. Thus, all sampled nests contained newly emerged adults and their mother (Flores-Prado et al. 2008). All collected nests exhibited characteristics of those nests that are used by first time (Flores-Prado et al. 2008). Nest collection was performed early in the morning (8–10 am) to ensure that all members were inside the nest. The entrance of each collected nest was sealed with Teflon and masking tape to be transported to the laboratory for analysis.

### Bee features measurement

Individuals were withdrawn from the nests and sacrificed by freezing in order to perform morphometric measurements. Data related to body size were obtained using three morphometric measures: (i) intertegular width, which represents the distance between the points where the lobe of each forewing is inserted in the thorax (Snodgrass 1993); (ii) wing length, which corresponds to the distance from the base to the top of the left wing; and (iii) mandible area, estimated as the product of its height (measured from the lowest point at the base of the condylar ridge, to the upper point at the trimma) and its base length (measured from the apex of the condylar ridge to the lowest point detected at the base of the condylar ridge) divided by two. Both intertegular width and wing length have been described as proxies of body size (Cane 1987; Smith and Weller 1989; Bullock 1999; Flores-Prado et al. 2008); landmarks used to calculate mandibular area were identified from Michener and Fraser (1978).

The measurement of body features was taken using images obtained with a digital camera coupled to a stereomicroscope (Olympus SZ61n) and processed by the Mshot software. As the mother could not be distinguished from the female progeny, all females in the nests were measured. In order to determine whether there is a potential confounding effect of including one individual of the previous generation on the measurements, variance/mean ratios were calculated as an indication of the relative deviation of individual measures with respect to the nest's mean. As variance/mean ratios were low (0.015 ± 0.004 for intertegular width, 0.028 ± 0.006 for wing length, and 0.013 ± 0.008 for mandible area of bees from *C. quila*; and 0.008 ± 0.001 for intertegular width, 0.036 ± 0.013 for wing length, and 0.010 ± 0.005 for mandible area of bees from *R. ulmifolius*), mean values may be used as reliable body size measurements. The minimum number of offspring in *C. quila* nests was three individuals; therefore, at least four bees were present per nest including the mother. In the case of *R. ulmifolius*, only four nests contained 1 individual; hence, they were not considered in the calculations of variance/mean ratios.

### Measurement of nest features

After each nest was opened in the laboratory, two proxies of nest architecture were measured with a digital caliper (Mitutoyo digital caliper, 0.01 mm precision): (i) tunnel length, representing the maximum length of the nest and (ii) tunnel diameter, represented by the average of three measurements, one on each edge and one in the middle of the nest. Furthermore, wood density for each host was used as a proxy for hardness and hence boring difficulty. Wood density was estimated from ten random nests for each host species, using the method described by Niemeyer (2013).

### Phenotypic selection analysis

Relative fitness (*w*) was calculated as *W*_*i*_/*W*_mean_, where *W*_*i*_ represents the observed number of females in nest (i.e., the number of hatched eggs that survived to become adults minus one female corresponding to the mother), and *W*_mean_ represents the average number of offspring per nest in the sampled population. Aiming to quantify the expected phenotypic change associated with each trait, selection differentials were calculated for each trait *i* (*S*_*i*_) as the covariance between the relative fitness (*w*_*i*_) and the value of the trait, *S*_*i*_ = cov (*z*_*i*_, *w*). The measured traits were divided into two groups: (i) bee features: intertegular width, wing length and mandible area and (ii) nest features: tunnel diameter and length. Quadratic (i.e., squared values) and correlational (i.e., pairwise trait product) coefficients were calculated from raw trait values. All coefficients were standardized (to mean = 0 and variance = 1) in order to make them comparable.

Relative fitness and standardized coefficient values were analyzed using the Lande and Arnold (1983) equations, in order to calculate the linear (*β*_*i*_) and nonlinear (*γ*_*i*_) selection coefficients associated with each trait. As some data sets presented a non-normal distribution, the significance of coefficients was tested by a bootstrapping procedure with 5,000 iterations which generates a confidence interval that indicates significance at *P* < 0.001 when the confidence interval does not overlap zero (Jordano 1995; Weber and Kolb 2013). For those traits showing nonsignificant linear gradients, the minimum sample required (MSR) for achieving statistical significance at *α* = 0.05 was estimated as: MSR = (*tσ*/*β*)^2^, where *t* = 1.96, *β* = selection gradient estimate, and *σ* = selection standard deviation estimate (Medel 2000). The MSR calculations were performed to discard mathematical artifacts due to low sample sizes; as a thumb rule for this failure-safe method, sample size issues are considered to be negligible when MSR ≥ 2N or when MSR exceeds the actual size of the population (Johnston 1991; Medel 2000).

The data set (including bees nesting at both host plants) was first analyzed as a whole and then separate analyses were conducted for each host species. Aiming to compare selection gradients between hosts, an analysis of covariance (ANCOVA) was conducted which included significant trait values as covariates; *P* values were corrected for multiple comparisons using a sequential Bonferroni adjustment (Murúa et al. 2010). All analyses were conducted in R version 2.15 (R Development Core Team 2012), using the packages mgcv, car, and boot.

## Results

A total of 105 *M. postica* nests were collected, 58 from *Chusquea quila* and 47 from *Rubus ulmifolius*. Nests from *C. quila* contained 3 to 10 individuals, whereas those from *R. ulmifolius* contained 1 to 7 individuals. Stems of *C. quila* were significantly denser than those of *R. ulmifolius* (mean ± 1SE, *C. quila*: 0.26 ± 0.02 g/cm^3^; *R. ulmifolius*: 0.07 ± 0.01 g/cm^3^; *t* = 12.93, df = 9, *P* < 0.001).

**Figure 1:**
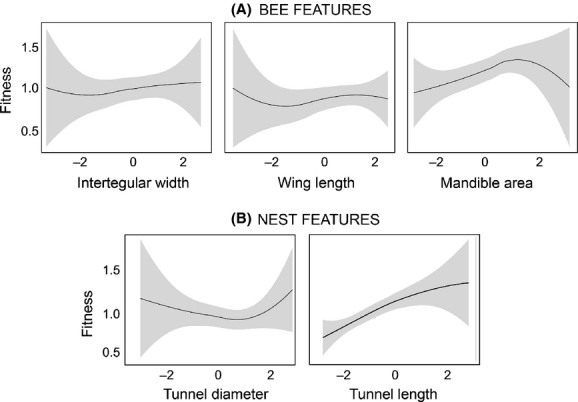
Relationships between relative fitness and measured traits (based on standardized values) for (A) bee features and (B) nest features.

Natural selection patterns were first analyzed on the whole population (i.e., comprising nests from both *C. quila* and *R. ulmifolius*); no significant linear or nonlinear selection coefficients were obtained for bee features. However, for nest features, a significant and positive linear selection gradient for tunnel length was found (Table 1; Fig. 1), indicating that those bees building longer nests have more offspring. None of the nonlinear gradients was significant. The failure-safe calculations indicated that at least 1,120–14,520 additional nests (exceeding the number of available nests at the study site) would be required to achieve statistical significance at *α* = 0.05 (except for mandible area, which would required at least 145 nests), indicating that the patterns observed are unlikely to be an artifact of a low sample size.

**Table 1:** Selection differentials (S_*i*_′), linear (*β*_*i*_′), and nonlinear (*γ*_*ii*_′) selection coefficients for bee and nest features (nests from *C. quila* and *R. ulmifolius* pooled together); standard errors are presented in parentheses

Trait *i*	*S*_*i*_′	*β*_*i*_′	*γ*_*ii*_′
Bee features			
Intertegular width	0.04	0.01 (0.06)^NS^	0.79 (1.30)^NS^
Wing length	0.04	0.01 (0.06)^NS^	0.16 (1.35)^NS^
Mandible area	0.10	0.10 (0.06)^NS^	−1.25 (1.13)^NS^
Nest features			
Tunnel diameter	−0.01	−0.03 (0.05)^NS^	0.78 (0.57)^NS^
Tunnel length	0.23	0.23 (0.05)*	−0.19 (0.25)^NS^

NS, not significant.

* Significant at *P* < 0.001 after bootstrapping procedures.

**Figure 2:**
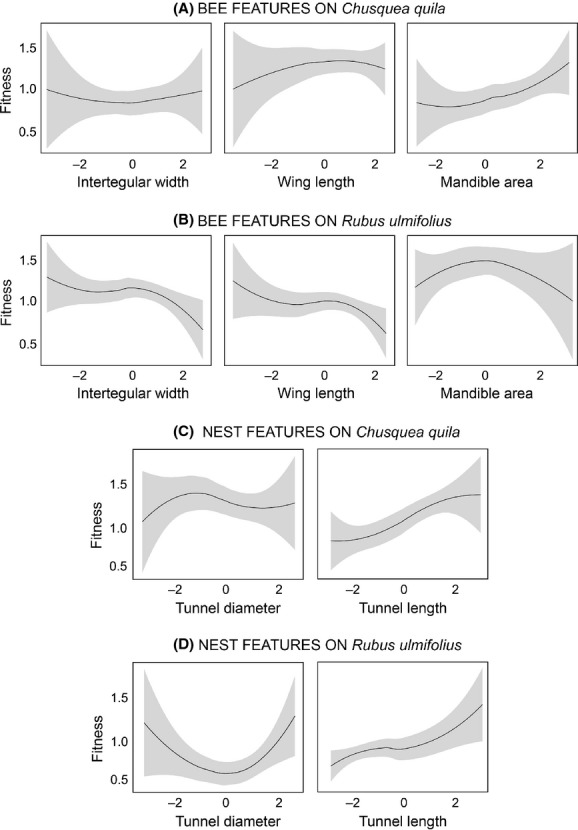
Relationships between relative fitness and measured traits (based on standardized values) for bee features in the nesting plants *Chusquea quila* (A) and *Rubus ulmifolius* (B), and nest features on *Chusquea quila* (C) and *Rubus ulmifolius* (D).

Then, analyzing selection gradients on each host plant separately, a positive and significant linear gradient was obtained for mandible area of bees from *C. quila*, a pattern that was not detected in bees from *R. ulmifolius*. Further, tunnel diameter showed a negative and significant linear gradient for bees from *C. quila*, and tunnel length linear gradient was significant and positive for bees both from *C. quila* and *R. ulmifolius* (Table 2; Fig. 2). As found on the whole population analysis, none of the nonlinear gradients were significant, with the exception of the correlational gradient between tunnel diameter and length (*γ* = −1.21, SE = 0.57) in bees from *C. quila*, whose negative value suggests that bees build longer but narrower nests inside *C. quila* branches. Our failure-safe calculation for those nonsignificant gradients on bees from *C. quila* yielded MSR values between 8,000 and 8,020 additional nests required to achieve statistical significance, and for bees from *R. ulmifolius* MSR values ranged between 102 and 1,128 additional nests, indicating in both cases that low sample sizes are unlikely to be influencing the obtained results; hence, the detected selection patterns are robust.

**Table 2:** Selection differentials (*S*_*i*_′), linear (*β*_*i*_′), and nonlinear (*γ*_*ii*_′) selection coefficients for bee and nest features determined separately for each host, *C. quila* and *R. ulmifolius*; standard errors are presented in parentheses

Trait *i*	Host	*S*_*i*_′	*β*_*i*_′	*γ*_*ii*_′
Bee features				
Intertegular width	*C. Quila*	0.02	−0.01 (0.06)^NS^	0.80 (1.87)^NS^
	*R. ulmifolius*	−0.13	−0.09 (0.12)^NS^	−2.28 (2.21)^NS^
Wing length	*C. quila*	0.03	0.01 (0.06)^NS^	−1.13 (1.59)^NS^
	*R. ulmifolius*	−0.13	−0.09 (0.12)^NS^	0.07 (2.37)^NS^
Mandible area	*C. quila*	0.14	0.14 (0.06)*	1.57 (1.75)^NS^
	*R. ulmifolius*	0.01	0.04 (0.10)^NS^	−3.34 (1.60)^NS^
Nest features				
Tunnel diameter	*C. quila*	−0.03	−0.13 (0.06)*	−0.98 (0.83)^NS^
	*R. ulmifolius*	0.05	0.08 (0.09)^NS^	2.58 (0.85)^NS^
Tunnel length	*C. quila*	0.22	0.26 (0.06)*	0.16 (0.38)^NS^
	*R. ulmifolius*	0.23	0.25 (0.09)*	0.35 (0.43)^NS^

NS, not significant.

* Significant at *P* < 0.001 after bootstrapping procedures.

Comparing the selection results between hosts, we found that bees from *C. quila* presented higher fitness values (3.35 ± 0.21 new adult females from *C. quila* versus 2.32 ± 0.22 from *R. ulmifolius*; Table 3). Relative fitness was also influenced by the interaction between host and tunnel diameter and by tunnel length, the latter factor having the strongest effect on fitness, because bees that construct longer nests have greater fitness.

**Table 3:** Analysis of covariance of the impact of host and nest features on the relative fitness of *Manuelia postica*. Degrees of freedom (df), sum of squares (SS), and *F* values are presented

Source	df	SS	*F*	*P*-value
Host (H)	1	0.98	4.29	0.04
Tunnel diameter (D)	1	0.26	1.15	0.29
Tunnel length (L)	1	6.23	27.33	<0.01*
H x D	1	1.16	5.07	0.03
H x L	1	0.46	2.00	0.16
Error	99			

* Source of variation that retained significance after sequential Bonferroni correction.

## Discussion

Under a scenario of host-mediated feeding divergence, the strong association between plants and herbivores may lead to disruptive selection when insects change hosts (Feder et al. 2003; Nosil 2007). Under such conditions, host-specific assortative mating associations are expected to occur (Drès and Mallet 2002). Carpenter bees use host plants as nesting substrates and not as feeding sources. No evidence was found for disruptive selection on traits associated with the use of alternative hosts, that is, mandible area and nest architecture. However, the data showed positive and significant linear selection forces acting on mandibular area on bees from *C. quila*, which were not detected on bees from *R. ulmifolius*. Such pattern could be explained by considering the differences detected in wood density, *C. quila* being a more difficult host to build a nest in, but giving larger rewards in terms of fitness. Thus, a directional selection toward broader mandibles could be associated with the use of the host with the denser wood.

Herbivore insects should evolve increased preferences for higher quality or more abundant hosts (Fry 1996). Some carpenter bees do not show preference toward particular plant species as nesting substrates (Hurd 1978; Bernardino and Gaglianone 2008), but do show preferences for more abundant host plants (Bernardino and Gaglianone 2008). Previous observations show that *C. quila* is more abundant than *R. ulmifolius* in the study area (unpublished data), and the relative use of *C. quila* as a nesting substrate is higher than in *R. ulmifolius* (Flores-Prado et al. 2008). Additionally, the use of an alternative host plant can be explained if such species constitutes the ancestral host on which an insect has had high survivorship (Berlocher and Feder 2002); this is to be expected to be the case of the native host, *C. quila* but not for the alloctonous one, *R. ulmifolius*. It seems paradoxical that *M. postica* nests on *R. ulmifolius*, an alloctonous species and in which nesting results in lower fitness, even when the native host *C. quila* is more abundant than *R. ulmifolius* and shares with the bee a long-term evolutionary history; in this scenario, natural selection is expected to make *C. quila* the only host species for *M. postica*. The fact that the wood of *R. ulmifolius* is much softer than that of *C. quila* less dense by about one order of magnitude, thus constituting a better substrate for bee females to bore in, could be responsible for such pattern.

While host-related morphological differences have been documented in phytophagous insects, no clear adaptive significance regarding host utilization (Emelianov et al. 1995) has been demonstrated. Mandible morphological differences associated with materials used for nest construction by some bee species did not showed strong evidence for adaptive differences on mandible shape (Williams and Goodell 2000). However, a strong relationship between mandible shape and nest material type has been observed in wasps (Hansell 1987; Sarmiento 2004), suggesting that the evolution of mandible has been influenced by the nest construction and that many taxa may face a similar scenario with nest materials representing strong selection forces (Sarmiento 2004). In *M. postica*, the directional selection on mandible area in bees nesting in the harder host and the higher fitness attained on that host suggest that the adaptive value in mandibular area could be associated with the particular host used for nesting. Further, the present results show strong selection on nest features that directly determine the reproductive outcome of *M. postica*, being particularly relevant the length of the nest tunnel, which is directly related to the number of eggs laid. Further, this pattern was consistent in both host-plant species assessed. These findings suggest that the nests of *M. postica* could be considered as an extended phenotype, under the hypothesis that phenotypic variation on nests (e.g., shape, architecture, size) are directly related with allelic variation on carpenter bees, as galls are for galling insects (Dawkins 1982; Crespi and Worobey 1998; Inbar et al. 2010).

A negative correlation between the number of individuals found inside a nest (individuals that represent the progeny of a female) and their mean size (estimated by wing length and intertegular distance) has been previously described for *M. postica* (Flores-Prado et al. 2008). Additionally, a positive correlation between nest length and the number of individuals found inside of them has been also reported (Flores-Prado and Niemeyer 2012). A potential trade-off between body size and progeny is likely to occur if, for example, females have a fixed amount of resources available for reproduction (Flores-Prado et al. 2008). This kind of trade-off has been broadly described in semelparous arthropods that exhibit no parental care (related to continuous food provisioning), as is the case of *M. postica* (review: Fox and Czesak 2000). Despite the fact that nest length was significant and positively correlated with fitness for bees from both *C. quila* and *R. ulmifolius* (supporting previous evidence that bees building longer nests have more offspring), no negative correlational gradients between bee size and nest length were found, as could be expected based on previous reports (Flores-Prado et al. 2008). Thus, no support was found for a possible trade-off as described above, shaped by natural selection operating on bee body size and nest length.

Although it has been proposed that several bee species do not extensively use their mandibles (Berlocher and Feder 2002), carpenter bees are characterized by powerful mandibles, due to their importance in nest construction (O'Toole and Raw 1991). Carpenter bees are included in the Megachilidae tribe Lithurgini (O'Toole and Raw 1991) and the Apidae tribes Xylocopini, Ceratinini (Michener 2007), and Manueliini (Flores-Prado et al. 2010). Several similarities in mandibular structures between species belonging to these tribes have been reported, which have been interpreted as convergences (Michener and Fraser 1978). Interestingly, species from these tribes construct nests using woody materials (twigs or tree branches), and nest construction has been hypothesized as a trait that has evolved independently (O'Toole and Raw 1991). In that sense, carpenter bees emerge as a valuable model to address inter- and intraspecific phenotypic variation in traits associated with alternative host use and which directly affect fitness (e.g., mandibles and nest architecture), and to explore the shape and intensity of natural selection acting upon such traits.
